# Development of a footwear sizing system for diabetic feet

**DOI:** 10.1016/j.heliyon.2024.e37824

**Published:** 2024-09-11

**Authors:** Bibhu Dash, Md. Rayhan Sarker, Md. Mukter Alam, Asack Mamun Asick, Aklima Begum

**Affiliations:** a Institute of Leather Engineering and Technology, University of Dhaka, Dhaka, 1209, Bangladesh; b Electronics Division, Atomic Energy Centre, Dhaka, 1000, Bangladesh; c Texas State University, San Marcos, TX, 78666, USA

**Keywords:** Diabetic patients, K-means clustering, Footwear sizing system

## Abstract

The number of diabetic patients is increasing rapidly who have vulnerable feet and might be easily affected by different adversities. Since there is no available footwear sizing system for diabetic patients, manufacturers produce diabetic footwear of different sizes and fittings based on other available footwear sizing systems, which may result in inappropriate fitting. To get footwear with proper fittings, diabetic patients may go for customized or bespoke footwear based on their foot conditions, which is very costly. This study attempts to explore the foot complications of diabetic patients and categorize their feet to create a new sizing system using foot measurements from 102 male diabetic patients based on three dimensions of human feet, namely foot length, ball girth, and instep circumference. K-means data clustering is followed to categorize the data into three broad groups, namely small, medium, and large groups for footwear sizing. The developed footwear sizing system uses a sizing interval of 8 mm and a fitting interval of 6 mm. This study suggests a total of 11 sizes along with 24 different fittings for the footwear manufacturers for producing diabetic footwear. This newly developed footwear sizing system has a total of 79.41 % coverage where there are 10, 10, and 4 fittings in the small, medium, and large groups, respectively. The proposed footwear sizing system can help footwear manufacturers understand the proper size and fit of diabetic patients’ feet so that they can make appropriate footwear for diabetic patients economically.

## 1 Introduction

Humans have been wearing shoes for almost 30,000 years [1]. Despite its origins as a defensive coating for the feet, current footwear is meant to serve a variety of functions, which is evaluated for success according to three aspects: form, function, and fit, while the last one is the most crucial factor in determining function and form when it comes to footwear [2]. While the ability of footwear to fulfill its primary purpose, such as feet protection, is referred to as function and the ability of footwear to adapt the morphology of feet appropriately is referred to as fit [3]. Poor fitting footwear may lead to several foot complications, such as blisters and ulcers [4]. When footwear is overly tight, tissue compression is caused by the interface pressure, which is painful for the wearer [5]. Conversely, if footwear is excessively loose, the wearer will experience foot slippage from the footwear [6]. Thus, properly fitted footwear plays a vital role in ensuring favorable feet health. Despite this, most people are indifferent to wearing footwear of correct sizes and fittings. According to a study by Burns et al. [7], about 63–72 percent of participants wore shoes that were not properly fitted. A previous study conducted by Alpert [8] showed that one in every three aged male participants has experienced foot related problems such as pain, stiffness, or achiness because of foot sickness and wearing improper fitting footwear. Most importantly, for diabetic patients, wrong sized and ill-fitted footwear increases the risk of severe injuries such as foot ulcers, which may lead to foot ulcerations [9]. Meanwhile Reiber et al. [10] found that foot problems such as ulcers, corns, ankle injuries, chronic discomfort, and blisters increase when inappropriate footwear is used by diabetic patients. Therefore, it is imperative to wear proper size and well-fitted footwear for diabetic patients.

Approximately 573 million individuals globally suffer from diabetes in 2021, with the majority of these individuals living in low- and middle-income countries [11,12]. In these countries, diabetes is directly linked to over 1 million deaths per year, and both the incidence and prevalence of the disease have been continuously rising over the past few decades [13]. In Bangladesh, 8.4 million adults had diabetes in 2019 and it is expected to nearly double (15.0 million) by 2045 [14,15]. Studies, such as a systematic review and meta-analysis, as well as results from national surveys, revealed that the prevalence of diabetes among adults in Bangladesh has significantly increased from 5 % to 14 % from 2001 to 2017 [16]. In a study in Bangladesh found that many diabetic patients didn't wear properly fitting footwear and consequently, it was contributing to several foot complications such as hammer toes, hallux valgus deformity, and foot pain [17].

A particular footwear (shoe) sizing system is used to manufacture footwear, which ensures proper size and fit for a group of people. Numerous shoe sizing systems are in use all over the world, such as the UK shoe size, the USA shoe size, the Paris point shoe size, the Japanese shoe size, and the Mondo point shoe size. There were a few studies on the development of shoe sizing scales for men and women or a specific group of people. For example, Limon et al. [18] developed a shoe sizing scale for the women of Bangladesh; Lee et al. [19] developed a shoe sizing system for Taiwanese females; Shariff et al. [20] proposed a new shoe-sizing system for Malaysian women using 3D foot scanning technology; Kim & Do [21] manifested a footwear sizing system for elderly men's based on their foot shapes; and Wannop et al. [22] formulated a footwear sizing system for the athletes of a national football league. However, till now, no particular shoe sizing system is available for diabetic patients around the world. On the other hand, male participants have larger feet than female participants [23], and female participants have larger feet breadth than those of males' [24], the shape and size of the diabetic feet may vary from person to person. Moreover, diabetic patients either wear customized shoes that are very expensive or buy usual footwear, which is mostly inappropriate for their feet. So, it is necessary to develop a particular shoe sizing system for diabetic patients to ensure proper size and fitting as well as to produce diabetic footwear on a mass scale basis at a reasonable price. Hence, this study aims to develop a footwear sizing system for male diabetic feet. For this purpose, this study uses the manual method for data collection from male diabetic patients in the context of Bangladesh. Several studies used a manual approach to measure foot dimensions directly for developing a foot size information system for shoe last design, foot shape analysis of adult males [25], children [26], shoe sizing systems for women [18], while other studies used 3D scanning technologies to measure foot dimensions for adults and children [27,28], elderly men's [21], and various age groups [29,30]. In a few studies, a 4D scanning system was used for dynamic foot morphology examination [31], dynamic foot shape measurements [32]. However, foot 4D models possess some constraints. Their absence of external validity is the first issue. Second, assumptions and simplifications such as linear material properties, simplified foot shape and structure, the absence of friction and slip boundary conditions, etc. are common challenges in foot 4D models (A. [33,34]). In the case of laser-scanning based 3D foot shape assessment devices are expensive in nature (Y. [35]; A. [33]; A. [36]). Moreover, the different weight imparted when the sole meets the floor during 3D scanning makes it unsuitable to view merely the foot surface shape in the 3D data [37]. Thus, a practical and cost-effective manual method has been adopted for this study. Manual foot measurement methods provide distinct benefits compared to 3D measurement approaches. Although 3D foot scanning offers in-depth information and enables precise evaluation of foot shape [38], manual measurements have their own advantages in specific areas. Compared to 3D technologies, manual foot measurement gives more accuracy and dependability [39]. Using manual foot measures reduces the impact of inconsistencies generated by new measurement technologies, leading to more consistent and dependable outcomes [40]. Furthermore, employing manual approaches may offer a more direct and simpler approach, hence minimizing the chances of measuring errors associated with the skills of experts or intricate algorithms utilized in 3D scanning software and rendering them a beneficial instrument in diverse fields such as clinical practice and the footwear industry [38,40]. In addition, Witana et al. [41] established that the differences between simulation methods and manual methods are insignificant. Against this backdrop, this study follows a manual method for foot measurement to develop a footwear sizing system for diabetic feet in Bangladesh, along with the following objectives:

(1) To explore the current state of diabetic patients in Bangladesh.
(2) To measure diabetic patients' feet by a manual method.
(3) To develop a footwear sizing system for male diabetic feet.

In this study, correlation analysis and K-means cluster analysis are performed to develop the footwear sizing system for diabetic patients. The novelty and contributions of this study are manifold. Firstly, this study is a totally new attempt where a novel footwear sizing system is developed for male diabetic feet in the context of Bangladesh. Secondly, a manual protocol of foot measurement for footwear sizing development is introduced. Thirdly, a closer examination of diabetic foot problems in Bangladesh is revealed. Finally, implications of this study in real-life settings are provided.

## 2 Methodology

### 2.1 Research design

**Fig. 1:**
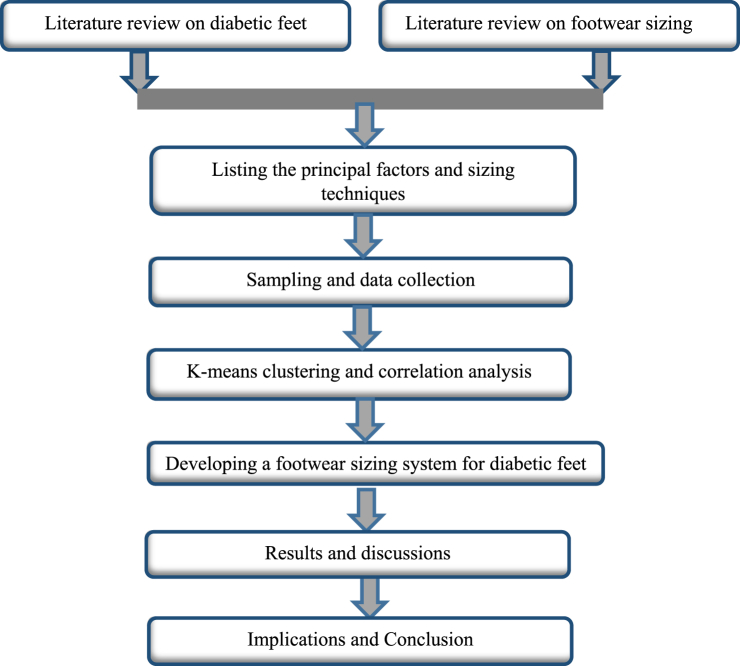
Research framework of this study.

The primary goal of this research is to develop a footwear size system for male diabetic feet in the context of Bangladesh. The relevant variables for developing a footwear sizing system were identified from the literature review. Twelve variables were identified from the literature review. Finally, three variables (i.e., foot length, ball girth, and instep circumference) were selected to develop the sizing system. These three variables were used to categorize the data into three main groups using K-means cluster analysis, and the data were also used for correlation analysis and significance tests (i.e., p-value). The research framework used in this study to select the most principal variables to develop the footwear sizing system for diabetic patients is delineated in Fig. 1.

### 2.2 Sampling and selection of prime factors

Proper selection of principal components is a prime condition for any survey-based research study. In this study, we considered the most significant foot dimensions, i.e., foot length, ball girth, and instep circumference, for foot measurement as suggested by Shariff et al. [20], to avoid complexity in footwear sizing system development. A simple random sampling was used for data collection. The probability sampling technique, known as simple random sampling (SRS), involves selecting participants from a population randomly. The likelihood of being chosen is the same for every member of the population, and this technique usually results in representative and impartial sampling [42]. We set some inclusion and exclusion criteria for choosing diabetic patients in this study. Both Type 1 and 2 diabetic patients were included in this study. However, a prior close foot examination was performed to consider whether a diabetic patient should be included in the study or not. We excluded the diabetic patients who required customized footwear due to serious foot complications (e.g., foot ulcer and amputation). This judgement was conducted by the research group based on their expertise.

### 2.3 Solution methodology

#### 2.3.1 Correlation analysis

Correlation is an analysis that measures the degree of association between two variables and the direction of the relationship [43]. Pearson's correlation, also referred to as Pearson's R, is a correlation coefficient that is widely utilized in linear regression. In order to determine how closely related two sets of data are, correlation coefficient formulas are used. Correlation analysis using Eq. (1) was used in various research studies to determine the relationship between different parameters of foot [44]. Therefore, in this study, correlation analysis was used to assess the control variables for developing the footwear sizing system for diabetic feet.

(1) $$r=\frac{n\sum \text{xz}-\sum x\sum y}{\surd \left[n\sum x^{2}-{\left(\sum x\right)}^{2}\right]\surd \left[n\sum y^{2}-{\left(\sum y\right)}^{2}\right]}$$

Here, x and y are the two variables, and r is the correlation coefficient.

#### 2.3.2 Significance test (p-value test)

The p-value is a useful tool for assessing the significance of a test result in relation to the null hypothesis [45]. A statistical hypothesis known as a ‘null hypothesis’ asserts that no statistical significance can be found in a particular set of observations, while a null hypothesis is directly opposed by an alternative hypothesis [46]. In other words, if one of the two hypotheses is correct, the other one must be false. The statistical significance level is usually expressed using the *p*-value, which has a range of 0–1. When the *p*-value is lower, the case for rejecting the null hypothesis is more compelling (Md. [47]).

#### 2.3.3 Cluster analysis

Based on the observed values of numerous variables for each individual, the cluster analysis technique is used to divide similar observations into several groups [48]. Cluster analysis is a data-processing statistical approach, and selecting its algorithm is very crucial. It operates by classifying items into groups, depending on how strongly linked they are [49]. One of the most appropriate clustering techniques is the K-means technique. The k-means technique identifies clusters by locating their centroid points. The average of all the data points in the cluster is referred to as the centroid point. Every point in the dataset can be categorized into a group by repeatedly calculating the Euclidean distance between them. The centroid points are initially chosen at random and fluctuate while the algorithm is run [50]. In this study, K-means clustering was used to cluster the data into several footwear size groups.

### 2.4 Data collection and analysis

#### 2.4.1 Data collection

**Fig. 2:**
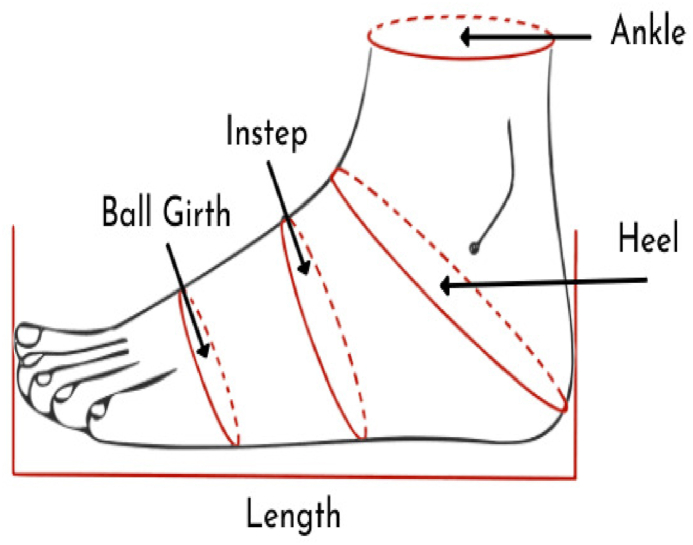
Graphical view of foot length, ball girth, and instep circumference.

Data were collected through a face-to-face survey based on a questionnaire following a developed survey protocol. In this study, first, a questionnaire was prepared to investigate different foot problems experienced by diabetic patients. The questionnaire format is provided in the supplementary file. Three dimensions of foot, namely foot length, ball girth, and instep circumference, were measured carefully using a foot length measuring device, measuring tape (8 mm), calipers, and pencil. The definitions of these foot dimensions are given in Table 1, and their graphical view is shown in Fig. 2. Foot dimensions were measured directly using a manual method described by Mauch et al. [26]. In this study, three trained enumerators measured all anthropometric data of 102 male diabetic patients to ensure maximal data reliability. The data from the participants were collected three times to ensure accuracy and reliability. Moreover, foot dimensions were measured while the patients were in standing positions to accommodate for variations in parameters because of the outspread of feet upon bodyweight acceptance [51]. While measuring the foot dimensions, some reference points were carefully observed and taken into account. Foot length is dictated by two reference points, i.e., the extremity of the heel and the tip of the longest toe. The first and fifth metatarsophalangeal joints are used as the reference points for measuring ball girth. Alternatively, middle cuneiform bone is used as the reference point for measuring instep circumference.

**Table 1:** Definitions of foot dimensions adopted in this study.

Term	Definition	Source
Foot Length (FL)	The measurement of the distance between the heel and the tip of the longest toe along a straight line.	Witana et al. [41]
Ball Girth (BG)	It is the foot's circumference covering the top of the first metatarsal bone, the medial margin of the head of the first metatarsal bone, and the lateral margin of the head of the fifth metatarsal bone.	Witana et al. [41]
Instep Circumference (IC)	The measurement of the smallest girth around the median cuneiform prominence.	Witana et al. [41]

(Source: https://www.poyter.com/how-to-measure-your-shoe-size/).

#### 2.4.2 Characteristics of sample

**Fig. 3:**
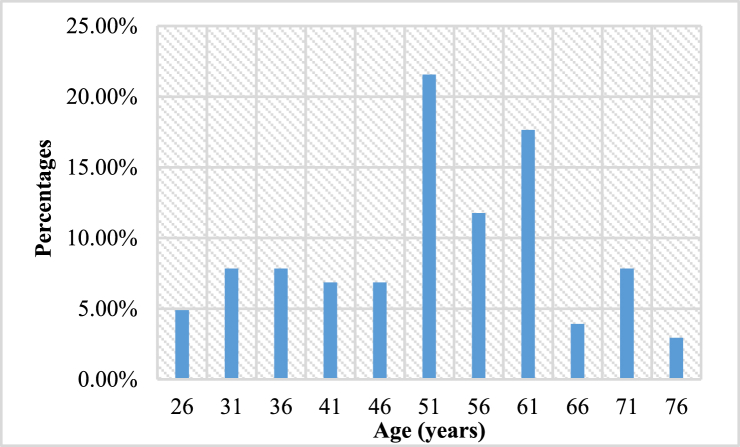
Percentages of diabetic patients of various age ranges.

Data were collected from 102 male diabetic patients who ranged in age from 26 to 80 years old with a mean value of 54.33 ± 13.18 years. The height range was from 134.62 to 185.42 cm with a mean value of 165.12 ± 8.98 cm. Fig. 3 shows the percentages of sample diabetic patients of various ages used in this study. The mean age of the participants was 54.33 ± 13.18 years. Here, it is seen that the highest number of patients about 51 % of the total participants were 51–65 years old.

### 2.5 Correlation analysis

**Fig. 4:**
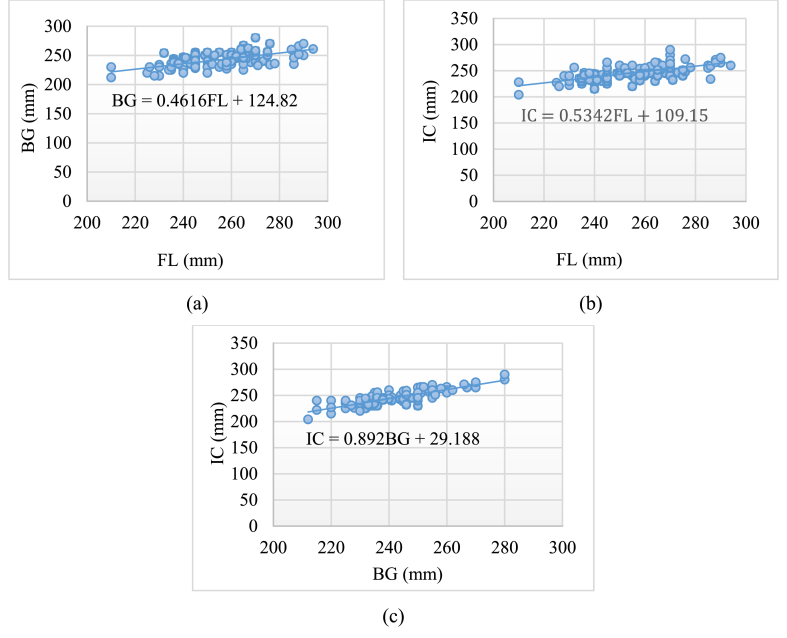
The scatterplots of (a) ball girth against foot length (b) instep circumference against foot length, and (c) ball girth against instep circumference.

Correlation analysis was applied to measure the correlation coefficient using Eq. (1) to determine the linear relationship between two variables and to define the strength of the relationship. A significance test (*p*-value) was also done to determine whether the result was significant or not. To assess linearity, scatterplots of ball girth, instep circumference against foot length, and ball girth against instep circumference, and the regression lines were plotted, which are shown in Fig. 4. The correlation equations, adjusted R^2^ values, regression coefficient (R), significance of each equation (F), P-values, and standard error by ANOVA test results are shown in Table 2, which shows how foot length, ball girth, and instep circumference are related in a way. The foot length shows a connection, with both ball girth and instep circumference with correlation coefficients of 0.60 and 0.65, respectively. Similarly, ball girth and instep circumference show a relationship with a correlation coefficient of 0.83. The high values of the correlation coefficients indicate strong links between the variables approaching or surpassing 0.80. Furthermore, all correlations have p-values <0.001 which indicates a connection between each pair of variables and is statistically meaningful.

**Table 2:** Correlation for ball girth, instep circumference against foot length, and ball girth against instep circumference.

Parameters	Length and ball girth	Length and instep circumference	Ball girth and instep circumference
Correlation Equation	BG = 0.4616FL + 124.82	IC = 0.5342FL + 109.15	IC = 0.892BG + 29.188
Multiple R	0.60	0.65	0.83
R^2^	0.36	0.42	0.69
Adjusted R^2^	0.36	0.42	0.69
Standard Error	11.13	11.36	8.30
ANOVA			
Significance F	1.94943E-11	1.2716E-13	2.54539E-27
P-value	<0.001	<0.001	<0.001

#### 2.5.1 Correlation matrix between the factors

The correlation matrix is a statistical technique used in multivariate analysis to examine the interactions and associations between variables. It includes pairing variables and assessing the strength of their connections using correlation coefficients [52]. A correlation matrix was applied in this study to measure the correlation coefficient between factors, such as foot length, ball girth, instep circumference, age, and height, and to figure out how strongly these variables are correlated with each other. Three colors were used for conditional formatting of the correlation matrix, where rose color was used for value −1, white color was used for value 0, and blue color was used for value 1. Here, if the values are close to 1, the color turns into bluish, while the values are near to 0, the colors tend to turn into white; and while the values are close to −1, colors also tend to turn into reddish (see Table 3). The correlation coefficient value between foot length and ball girth, foot length and instep circumference, and ball girth and instep circumference showed that they were statistically significant. While, the correlation coefficients between age and other factors (height, foot length, ball girth, and instep circumference) are negative and close to 0. The reason behind the result is that the physical growth of humans continues to increase till age 26 years and then falls gradually [53].

**Table 3:**
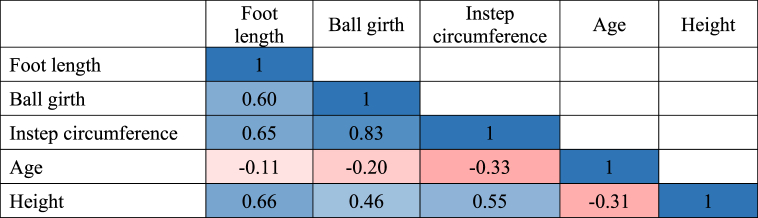
Correlation matrix among the factors.

### 2.6 Cluster analysis

**Fig. 5:**
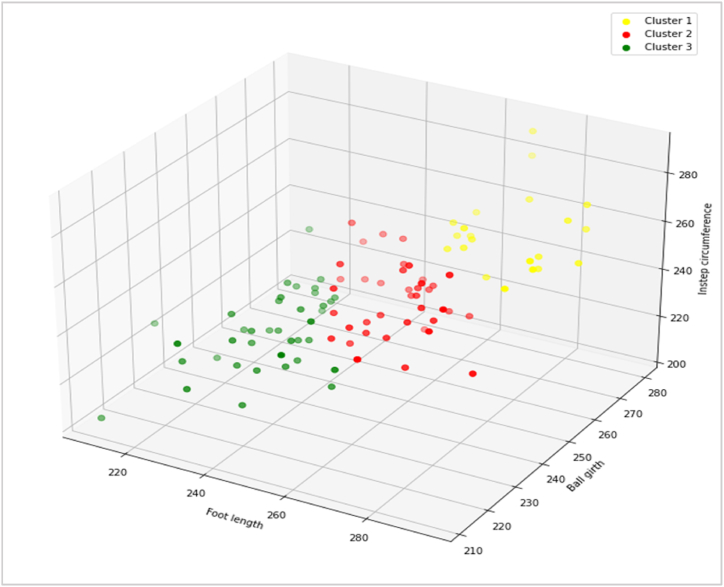
3D visualization of K-means clustering for three dimensions of foot.

Cluster analysis was applied to categorize the data into a number of groups, based on how closely they are related to developing an initial footwear sizing system. Clustering was performed based on selected factors: foot length, ball girth, and instep circumference. Cluster analysis resulted in three major groups named cluster 1, cluster 2, and cluster 3, which represent small, medium, and large groups, respectively. Each cluster or group contains a mean value, standard deviation, minimum value, and maximum value of foot length, ball girth, instep circumference, age, and height of that group. A k-means clustering algorithm was performed, which identifies clusters by locating their centroid value to group the data into three clusters, i.e., small, medium, and large. These clusters are represented by 3D visualization as shown in Fig. 5.

Table 4 presents the findings for cluster 1, cluster 2, and cluster 3. Cluster 1 represents the small group where the mean foot length is 237.71 ± 9.94 mm, the mean ball girth is 232.71 ± 9.89 mm, the mean instep circumference is 233.37 ± 10.02 mm, the mean age is 56.68 ± 14.66 years, and the mean height is 158.15 ± 9.24 cm. Cluster 2, which is classified as a medium-sized group, comprises various anthropometric measurements. In this cluster, the foot length, ball girth, instep circumference, age, and height have a mean value of 261.93 ± 9.32 mm, 243.09 ± 8.44 mm, 247.53 ± 8.43 mm, 55.14 ± 11.61 years, and 168.17 ± 4.72 cm, respectively. On the other hand, for cluster 3 (large group), the mean values were 277.33 ± 10.42 mm, 261.57 ± 8.93 mm, 265.52 ± 8.71 mm, 48.43 ± 12.52 years, 171.51 ± 7.17 cm for foot length, ball girth, instep circumference, age, and height, respectively.

**Table 4:** Cluster analysis for diabetic footwear sizing system.

Variables	Cluster 1				Cluster 2				Cluster 3			
	Mean	Standard deviation	Minimum	Maximum	Mean	Standard deviation	Minimum	Maximum	Mean	Standard deviation	Minimum	Maximum
*Foot length (mm)*	237.71	9.94	210	258	261.93	9.32	245	286	277.33	10.42	264	294
*Ball girth (mm)*	232.71	9.89	212	254	243.09	8.44	225	255	261.57	8.93	246	280
*Instep circumference (mm)*	233.37	10.02	204	256	247.53	8.43	230	266	265.52	8.71	251	290
*Age (year)*	56.68	14.66	30	80	55.14	11.61	26	76	48.43	12.52	32	72
*Height (cm)*	158.15	9.24	134.62	175.26	168.17	4.72	157.48	180.34	171.51	7.17	157.48	185.42

## 3 Results and discussions

The three measurements (foot length, ball girth, and instep circumference) were used to establish the basic parameters for developing a footwear sizing system for diabetic patients. The correlation study revealed that the instep circumference was more strongly related to foot length than the ball circumference (r = 0.60). In both situations, the p-value was <0.001, which suggests that the correlation coefficient was significant in both circumstances. There was a high relationship between ball girth and instep circumference, as measured by the correlation value r, which was 0.83. Additionally, circumferential measurements such as ball, waist, and instep have strong correlations among themselves, as well as with other measurements, with the exception of foot length [54]. However, ball girth is the only circumference measurement that cannot be adjusted with shoelaces, and there is no proportionality between the increase in foot length and ball girth [55]. Thus, foot length and ball girth were used as variables to develop the intended footwear sizing system for diabetic patients, offering several fittings for a particular footwear size.

**Fig. 6:**
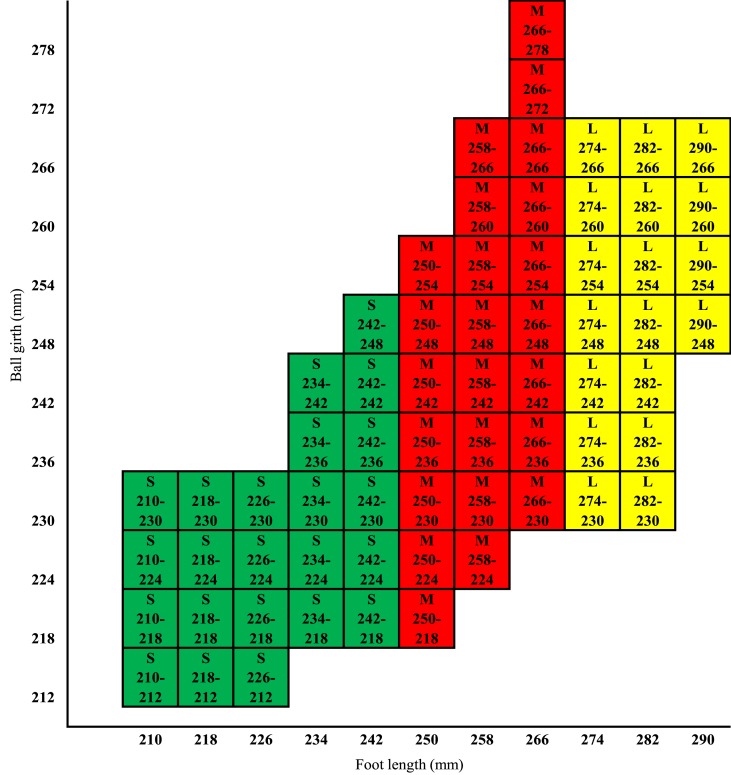
Derived footwear sizing scale for diabetic patients from K-means clustering.

One of the prime conditions for developing a footwear sizing system is to set the sizing interval. To avoid any confusion, it is preferable to create a footwear sizing scale that is compatible with the global footwear size standard (Y. [56]). Therefore, the developed footwear sizing system was created using the following criteria: foot length with an 8 mm interval and ball circumference with a 6 mm interval. The sizing system has three main groups, such as small, medium, and large groups, with a total of 11 sizes according to length, and 65 fittings according to ball girth (see Fig. 6). The total coverage of the shoe sizing scale is 98.04 %, where the small group has a coverage of 35.29 % with 23 fittings, the medium group has a coverage of 48.04 % with 24 fittings, and the large group has a coverage of 14.71 % with 18 fittings (see Table 5).

**Table 5:** Summary of footwear sizing system.

Terms	Foot type		
	Small	Medium	Large
Foot length (mm)	237.71	261.93	277.33
Ball girth (mm)	232.71	243.09	261.57
Scope of length (mm)	<250	250–273	>273
Number of sizes	5	3	3
Number of fittings	23	24	18
Coverage	35.29 %	48.04 %	14.71 %

**Fig. 7:**
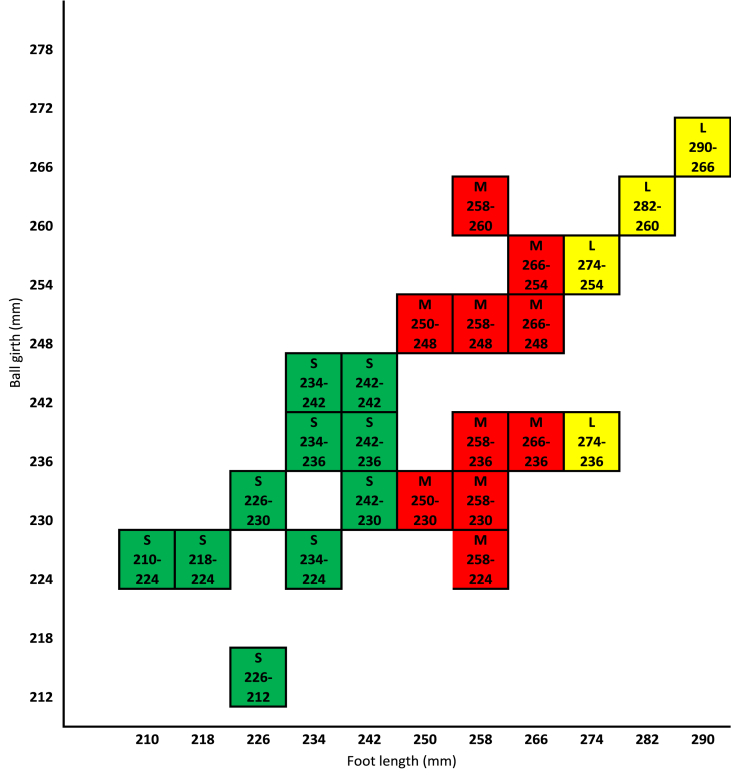
Suggested footwear sizing system for diabetic patients.

Subsequently, in order to reduce the number of fittings, maximum coverage fittings were identified and suggested along with 11 sizes (see Fig. 7). Then, the total coverage of the suggested footwear sizing system is 79.41 %, where the small group has a coverage of 30.39 % with 10 fittings, the medium group has a coverage of 38.24 % with 10 fittings, and the large group has a coverage of 10.78 % with 4 fittings (see Table 6).

**Table 6:** Number of sizes and coverages of suggested footwear sizing system.

Terms	Foot Type			
	Small	Medium	Large	Total
Number of sizes	5	3	3	11
Number of fittings	10	10	4	24
Coverage	30.39 %	38.24 %	10.78 %	79.41 %

There are various types of footwear sizing systems that are based on the stick length (usually 2 sizes larger than foot length), such as French, American, and British systems. Alternatively, some footwear sizing systems are based on the actual length of the foot, such as the Japanese, Chinese, and Mondo Point systems [57]. In this research, the footwear size system was based on the participant's actual foot length, and ball girth where S226-230 denotes a size appropriate for small groups, whose foot length falls between 226 and 233 mm and whose ball girth falls between 230 and 235 mm; M250-230 signifies a size appropriate for medium groups, whose foot length falls between 250 and 257 mm and whose ball girth falls between 230 and 235 mm; and L274-236 indicates that the size is suitable for large groups, whose foot length falls between 274 and 281 mm and whose ball girth measures between 236 and 241 mm.

There are three primary factors to consider when evaluating an efficient sizing system, i.e., the coverage, the number of sizes, and the level of fitness [19]. Though there is no exact sizing scale for diabetic patients, in this study, the findings of the suggested footwear sizing system for diabetic patients were compared with the results of the prior studies (see Table 7). A study conducted by Lee et al. [19] in Taiwan resulted in the development of a shoe sizing scale specifically designed for women. This scale consisted of 14 different sizes, 80 different fittings, and achieved a coverage rate of 92.70 %. There were a total of 8 fittings for 15 different sizes for Malaysian women [20]. Limon et al. [18] introduced a shoe sizing system with 27 size-fit combinations specifically designed for women in Bangladesh. In our study, the sizing method was designed based on K-means clustering and correlation analysis. This approach offered a minimum number of size-fit combinations with adequate coverage in comparison to prior studies. Moreover, it will help to reduce the complications of size-fit combinations. Thus, this study suggested a footwear sizing system that includes 11 sizes and a total of 24 fittings with a coverage of 79.41 %. The overall coverage has decreased because of the irregular sizes and shapes of the feet of the participants. This study did not aim for 100 % coverage due to the greater variety of sizes and fittings involved, which will lead the sizing system to customization.

**Table 7:** A comparison between previous studies and proposed footwear sizing system.

Terms	Lee et al. [19]	Shariff et al. [20]	Limon et al. [18]	This Study
	Total	Total	Total	Total
Number of sizes	14	15	9	11
Number of fittings	80	8	27	24
Foot measuring parameters	Foot Length (FL), Joint Width (JW), Arch Height (AH)	Foot Length (FL), Joint Width (JW), Joint Girth (JG)	Foot Length (FL), Joint Girth (JG), Joint Width (JW), Arch Length (AL)	Foot Length (FL), Ball Girth (BG)
Coverage	92.70 %	not found	JG 94.98 %, AL 98.77 %, and JW 88.02 %	79.41 %
Country	Taiwan	Malaysia	Bangladesh	Bangladesh
Gender/Purpose	Women	Women	Women	Diabetic male patients

The suggested shoe sizing systems can be used only for pre-diabetic and on-diabetic patients who do not have severe foot complications. Pre-diabetic and on-diabetic shoes can prevent further foot complications in diabetic patients. On the other hand, diabetic patients who have severe foot disorders (e.g., foot ulcers, hammer toes, etc.) require custom-made shoes based on their foot conditions; in this regard, no sizing system is applicable. For custom-made footwear, diabetic patients’ feet are modeled to find out their footwear sizes and fittings.

From the analysis of the collected data during the survey, this study also revealed that the majority of the diabetic patients were indifferent to their foot health; even they didn't know the relationship between foot health and footwear. Only 11.76 % of the participants knew that there is a relationship between foot health and footwear, and about 15.69 % of the total sample knew the size of the footwear that suits their feet. About 1.96 % of the total sample got suggestions from doctors on what types of footwear they should wear. Importantly, 25.49 % of the participants were facing different types of foot problems, where multiple foot problems and fungal infection of nails were significant with a percentage of 8.82 and 6.86, respectively (see Table A1 in the Appendix).

## 4 Implications of the study

The prevalence of diabetes is currently on the rise, resulting in an increased need for diabetic footwear. However, there is no footwear sizing system for diabetic patients in Bangladesh, which results in manufacturing footwear of different sizes and fittings as like regular footwear. Since diabetic patients have different foot dimensions, the sizes and fittings provided by the available retail footwear will not be able to ensure proper fit for diabetic patients. Moreover, diabetic patients are not serious about their footwear selection and their foot health. Most of them do not even know whether there is any relationship between their foot health and footwear or not. In this context, this study can guide the footwear manufacturers to know about the appropriate sizes and shapes of the feet of diabetic patients and to manufacture footwear of appropriate sizes and fittings for them. In addition, the proposed footwear sizing system may help diabetic patients to purchase the right footwear for themselves at a minimum cost rather than costly bespoke footwear.

## 5 Conclusion

This study used K-means clustering and correlation analysis to develop a footwear sizing system that includes three broad groups, such as small, medium, and large. The developed footwear sizing system provided a coverage of 79.41 %, with 10 fittings in the small group, 10 fittings in the medium group, and 4 fittings in the large group, along with 11 sizes. The major contribution of this research was to create a footwear sizing system for male diabetic patients, which was not investigated before in any other studies. This research not only provided the footwear industry with the opportunity with the opportunity to produce diabetic footwear of appropriate sizes and fittings to reduce the production cost but also provided scope for diabetic patients to purchase the right footwear at a minimum cost rather than customized footwear. Finally, this study may help to raise awareness among all diabetes-affected patients about their foot health and proper footwear selection.

The development of a footwear sizing system is a very complex issue. It is impossible to consider all aspects of a single footwear sizing system. Therefore, this study has some limitations that can be addressed in any future studies. This study considered only three dimensions of foot, i.e., foot length, ball girth, and instep circumference, to develop the sizing system, based on a previous study. However, the anthropometry of any diabetic foot may differ from regular foot. Therefore, principal component analysis could be conducted to overcome this limitation. Moreover, this study used the manual method for foot measurement, which has some limitations in terms of accuracy, consistency, and time efficiency. Hence, 3D and 4D scanners can be used in the future for modeling diabetic patients’ feet and subsequently for the development of a footwear sizing system.

## Ethics and consent section

This study didn't require ethical approval. We collected data from diabetic patients who didn't have vulnerable feet. Prior to the data collection process, we explained our study goals to the participants and took their approvals. During data collection, only three dimensions of foot such as length, width, and girth were measured carefully, which were not injurious to the participants. Therefore, the ethical statement was not necessary for this study.

## Data availability

All relevant data are included in this study.

## Funding

This research received no external funding.

## CRediT authorship contribution statement

**Bibhu Dash:** Writing – original draft, Methodology, Formal analysis, Data curation. **Md. Rayhan Sarker:** Writing – review & editing, Visualization, Validation, Supervision, Software, Resources, Project administration, Methodology, Investigation, Conceptualization. **Md. Mukter Alam:** Writing – review & editing, Visualization, Validation, Resources, Methodology, Investigation. **Asack Mamun Asick:** Writing – original draft, Methodology, Investigation, Formal analysis. **Aklima Begum:** Writing – review & editing, Software, Resources, Investigation, Formal analysis, Data curation.

## Declaration of competing interest

The authors declare that they have no known competing financial interests or personal relationships that could have appeared to influence the work reported in this paper.

## Figures and Tables

**Table A1:** No. of persons with different types of foot problems.

Types of foot problem	No of persons	Percentage (of total sample)
Fungal infections of nails	7	6.86 %
Calluses	2	1.96 %
Ingrown toenails	1	0.98 %
Dry skin	3	2.94 %
Blisters	1	0.98 %
Bunions	1	0.98 %
Allergy (itching)	1	0.98 %
Multiple problems (more than one)	9	8.82 %
Foot injury (surgery)	1	0.98 %
**Total**	26	25.49 %
