# Supplementary material for: Development of a footwear sizing system for diabetic feet
Source: Heliyon. 2024 Sep 11;10(18):e37824. doi: 10.1016/j.heliyon.2024.e37824 (PMC11422582; doi:10.1016/j.heliyon.2024.e37824)
Supplement: Multimedia component 1 [file mmc1.docx]

**Questionnaire form for the study on the development of a footwear sizing system for diabetic feet**

Before starting the survey, an enumerator will explain the study objectives to respondents. The enumerator will inform the respondents that the collected data will only be used for this research and publication; the data analysis report will be anonymous. Moreover, the enumerator will take verbal consent from the respondents before starting the survey. This study will only collect data from male diabetic patients who don’t have severe foot complications, such as ulcers, foot amputation, and neuropathy. To ensure proper data collection and avoid any kind of complexities, a medical officer will be included in the data collection team.

Serial no: Date:

Name : ……………………………………………………………………….………………

Address : ……………………………………………………………………………………….

Gender : …………………………….…… Age: ………………………….……………years

Phone: …………………………………….

1. Do you have full consent to take part in this survey? Yes No
2. Do you have diabetes? Yes No
3. How long are you facing diabetes? …………………………………
4. Do you know whether there is any relationship between foot health and footwear?

Yes No

1. Have you got any suggestions from doctor regarding footwear wear?

Yes No

If yes, then what: ………………………………………………………………………………………….

1. Do you know the size of footwear that suits your feet? Yes No
2. What types of footwear are you wearing?

……………………………………………………………............................................

…………………………………………………………………………………………

1. Have you ever faced any foot problems after diabetes? Yes No

If yes, then what:

|  | Bunions |  | Foot ulcers |  | Ingrown toenails |
| --- | --- | --- | --- | --- | --- |
|  | Hammer toes |  | Athlete’s foot |  | Morton toe |
|  | Plantar fasciitis |  | Metatarsalgia |  | Fungal infection of nails |
|  | Calluses |  | Corns |  | Blisters |
|  | Plantar warts |  | Dry skin |  | Flat foot (low arch) |
|  | Pes cavus (high arch) |  | Others |  |  |
|  | Others: ……………………………………………………………………………. | | | | |

1. What are the problems you are facing while wearing a pair of shoes?

………………………………………………..……………………………………………...……………………………………………………………………………………...

**Foot measurements:**

Foot Length : ……………………………………………………………… mm

Ball girth : ………………………….……………………………………mm

Instep circumference : .………...…………………………………………………… mm

| Signature of Enumerator |  | Signature of Respondent |
| --- | --- | --- |
